# La recherche pour la santé à Madagascar: état des lieux, défis et perspectives

**DOI:** 10.11604/pamj.2021.39.36.27462

**Published:** 2021-05-12

**Authors:** Joëlle Laure Sobngwi-Tambekou, Catherine Marie Jones, Clare Wenham, Michel Ratsimbason, Marie-Rolland Ratsimbazafy, Fidelis Adolphe Andriamizarasoa, Pamela Juma, Rhona Mijumbi-Deve, Justin Parkhurst

**Affiliations:** 1 London School of Economics (LSE) and Political Science, LSE Health, London, United Kingdom,; 2 Recherche Santé et Développement (RSD Institut), Yaoundé, Cameroun,; 3 London School of Economics and Political Science, Department of Health Policy, London, United Kingdom,; 4 Centre National d´Application de Recherches Pharmaceutiques, Antananarivo, Madagascar,; 5 Service d´Appui à la Recherche et à la Gestion des Connaissances, Direction de la formation et de la Recherche, Institut National de Santé Publique et Communautaire, Antananarivo, Madagascar,; 6 Direction des Études, de la Planification et du Système d'Information, Ministère de la Santé Publique, Antananarivo, Madagascar,; 7 Centre for Rapid Evidence Synthesis, College of Health Sciences, Makerere University, Kampala, Uganda

**Keywords:** Humains, priorités de santé, négociation, Madagascar, Humans, health priorities, negotiation, Madagascar

## Abstract

L´évolution et les défis contemporains de la recherche pour la santé (RS) à Madagascar sont peu documentés. Notre objectif est de faire un état des lieux du Système National de Recherche pour la Santé (SNRS) dans le but de comprendre les facteurs qui influencent son développement. Nous avons mené une étude de cas qualitative, qui consistait en une revue documentaire et des entretiens semi-structurés avec 38 informateurs clés. Nous avons effectué une analyse thématique et nous sommes inspirés des composantes du Baromètre des SNRS de l´OMS/AFRO pour structurer la présentation des résultats. À Madagascar, les institutions de RS opèrent sans un cadre législatif spécifique. Il existe toutefois un document de politique présentant les orientations nationales de la RS. Les ressources humaines sont insuffisantes, du fait de défis dans l´accès à la formation et la rétention des chercheurs. La collaboration internationale représente quasiment l´unique source de financement de la RS. Elle contribue également au renforcement des capacités humaines, institutionnelles mais ne favorise pas toujours l´adéquation entre la recherche effectuée et les besoins prioritaires du pays. Les efforts inaboutis de régulation et la modicité de l´investissement public dans la formation et la mise en œuvre de la recherche traduisent un engagement insuffisant des gouvernants pour la RS. La négociation de partenariats internationaux équitables, la disponibilité de financements publics, et l´alignement de la RS aux priorités nationales de santé constitueraient une base solide pour le développement du SNRS à Madagascar.

## Abstract (en)

The evolution and contemporary challenges of health research (HR) in Madagascar are poorly documented. We aim to gain insights on the factors that shape Madagascar's National Health Research System (NHRS) to better understand their influence. We conducted a qualitative case study, which included a documentary review and semi-structured interviews with 38 key informants. We carried out a thematic analysis and used the WHO/AFRO NHRS Barometer to structure the presentation of the results. There is no legislative framework to support HR activities and institutions. There is, however, a policy document outlining national priorities for HS. Human resources for HR are insufficient, due to challenges in training and retaining researchers. International collaboration is almost the only source of HR funding. Collaborations contribute to developing human and institutional capacity, but they are not always aligned with research carried out locally and the country's priority health needs. Incomplete efforts to improve regulation and low public investment in research training and research implementation reflect an insufficient commitment to HR by the government. Negotiating equitable international partnerships, the availability of public funding, and aligning HR with national health priorities would constitute a solid basis for the development of the NHRS in Madagascar.

## Introduction

La recherche pour la santé (RS) est une des pierres angulaires du développement et de la santé, sous condition qu´elle réponde aux besoins de connaissances sur les priorités définies par le pays et soit mise en œuvre avec des institutions nationales fortes et une masse critique de chercheurs locaux équalifiés [1].Faisant suite aux recommandations du Sommet Ministériel de l´Organisation Mondiale de la Santé (OMS) sur la RS de Mexico (2004), la rencontre internationale des leaders Africains sur la RS d´Abuja (2006) a marqué pour la première fois l´engagement officiel des gouvernements africains à optimiser leurs efforts en vue du renforcement des systèmes nationaux de recherche pour la santé (SNRS) [2]. Ces engagements internationaux ont été diversement implémentés dans les pays, conduisant à la coexistence sur le continent de pays disposant d´un SNRS mature, bénéficiant d´un financement public et de capacités locales solides, et de pays dans lesquels le système reste embryonnaire, en phase de construction [3-7].

En 2017, l´analyse situationnelle du SNRS à Madagascar effectuée par le Ministère de la Santé Publique (MSANP) a révélé des insuffisances en matière de coordination, de partenariat et de ressources humaines, financières et institutionnelles [8,9]. Cette analyse a été corroborée par une évaluation des performances des SNRS des pays de la région Afrique conduite par l´OMS (2018). Cette évaluation, basée sur des critères quantitatifs illustrant les différentes fonctions d´un SNRS, a révélé un score de performances de Madagascar parmi les plus bas (39% contre une moyenne régionale de 61%) [10,11]. En effet, la visibilité des chercheurs malgaches sur le plan international est faible. Pour l'année 2014, la productivité du pays en termes de publications scientifiques était de 188, soit 8 publications par million d'habitants et plus de 80% des articles étaient publiés en collaboration avec des chercheurs occidentaux, une situation qui reflète la dépendance du SNRS, des collaborations et financements extérieurs [12]. À Madagascar, les dépenses publiques de recherche et de développement représentaient 0,01% du produit intérieur brut (PIB) en 2017 [13], largement en deçà du taux d´1% du PIB préconisé par l´OMS et l´Union Africaine. Le soutien des partenaires scientifiques et financiers semble donc incontournable pour assurer le renforcement du SNRS. Toutefois, le leadership local à travers l´engagement des experts nationaux et la participation de l´ensemble des acteurs affectés ou impliqués sont indispensables pour faire avancer et pérenniser une RS en phase avec la priorisation des besoins par les experts locaux [14].

La stratégie de RS de l´OMS pour la région Afrique fournit des directives aux états membres pour renforcer leurs systèmes de recherche, mais pour atteindre les cibles données (en particulier celle du financement), ces interventions nécessitent l´engagement des gouvernements [15]. En effet, il est de la responsabilité des décideurs de faire de la recherche une priorité gouvernementale. Dans le but de comprendre les défis contemporains de la RS à Madagascar, nous avons procédé à un état des lieux du SNRS en insistant sur le rôle et l'importance des différents facteurs qui influencent son évolution.

## Méthodes

**Collecte des données**: les données de cette étude de cas proviennent d´une revue documentaire sur la RS à Madagascar et des entretiens effectués avec les informateurs-clés dans le pays. Cette étude de cas fait partie d´un projet qui avait pour objectif d´analyser les pratiques, les investissements ainsi que les obstacles et les facilitateurs du développement des SNRS en Afrique. La revue documentaire a recueilli des données issues d´articles scientifiques, de documents de politiques et de stratégies, et de rapports d´évaluations sur la RS à Madagascar. Un total de 30 entretiens ont été effectués entre le 04 mars et le 23 avril 2019 avec des acteurs gouvernementaux (n=8); des chercheurs et universitaires (n=14); des partenaires techniques et financiers (PTF) (n=16), sélectionnés après une cartographie des acteurs avec un chercheur local (Tableau 1). Ces entretiens, d´une durée moyenne de 30 minutes étaient réalisés par deux chercheurs, à l´aide d´une grille d´entretien semi-structurée. Ils portaient sur l´expérience des informateurs; leur perception du fonctionnement du SNRS avec ses forces, opportunités et points à améliorer; les exemples de bonnes pratiques; et enfin, les enseignements tirés de leur expérience. Les entretiens ont été enregistrés, transcrits, et sauvegardés sous format Word. Un consentement éclairé a été obtenu de tous les participants conformément aux directives éthiques en vigueur dans le pays. La clairance éthique a été obtenue du Comité d´Ethique de la Recherche de la « London School of Economics and Political Science » [CER 000757] et l´étude a été autorisée par le Comité d´Ethique de la Recherche Biomédicale de Madagascar (CERBM).

**Tableau 1:** liste des institutions visitées pendant l´enquête à Madagascar

Catégorie de parties prenantes	Institutions
**Universités**	Université d´Antananarivo
**Partenaires technique et financier/partenaires bilatéraux**	Ambassade de France
	CDC/USAID President´s Malaria Initiative (PMI)
	Institut de Recherche pour le Développement(IRD)
	Agence des Etats-Unis pour le Développement International (USAID)
**Partenaires technique et financier/organisations philanthropiques**	Fondation Mérieux
**Organisations internationales**	Banque Mondiale
	Délégation de la Commission Européenne
	Fonds des Nations Unies pour la population (FNUAP)
	Fonds des Nations Unies pour l´Enfance (UNICEF)
	Organisation Mondiale de la Santé
**Institutions publiques de recherche**	Centre National d´Application des Recherches Pharmaceutiques (CNARP)
	Centre National de Recherches sur l´Environnement (CNRE)
	Institut National de Santé Publique et Communautaire (INSPC)
	Institut national des sciences et technique nucléaires (INSTN)
	Institut Pasteur de Madagascar (IPM)
	Unité de recherche CHU Antananarive
**Gouvernement-ministères**	Ministère de l´Enseignement Supérieur et la Recherche Scientifique (MESRS)
	Ministère de la Santé Publique(MSP)
	Comité d´Ethique Biomédicale de Madagascar
**Gouvernement-structures autonomes**	Office National de Nutrition (ONN)
**Organisations Non Gouvernementales**	Agence Universitaire de la Francophonie
	CARE
	JHPIEGO
	PIVOT

**Analyse des données**: les transcriptions ont été importées dans le logiciel Dedoose pour un codage sur la base de thèmes conçus en équipe de façon déductive. Nous avons analysé les transcriptions en nous concentrant sur les éléments du SNRS en place et les processus qui facilitent ou entravent leur fonctionnement. Nous avons modifié nos codes à deux reprises avec des thèmes émergeant de façon inductive au cours du processus d´analyse. Les codages ont été comparés entre chercheurs pour en assurer la cohérence. L'amélioration du SNRS est fonction de plusieurs paramètres ; pour structurer et conceptualiser notre analyse, nous nous sommes inspirés des fonctions des SNRS du Baromètre de l´OMS/AFRO. Ces fonctions sont: le renforcement de la gouvernance, la création et le maintien des ressources humaines, la production et l´utilisation des connaissances, et le financement durable de la RS [7,10]. Les codes et les indicateurs mobilisés pour analyser chacune de ces fonctions sont présentés dans le Tableau 2.

**Tableau 2:** liste des codes et des indicateurs utilisés pour l´analyse des données, selon les différentes fonctions du SNRS à Madagascar

Fonctions	Codes et (sous-codes)	Indicateurs
**La gouvernance**	Gouvernance de la recherche pour la santé (institutions, législation, politiques, régulation, autre)	L´existence et la mise en œuvre d´une politique nationale, d´un plan stratégique, de textes règlementaires et des mécanismes de régulation de la recherche pour la santé tels que les comités d´éthique, ainsi qu´un mécanisme de coordination de la RS et un système de gestion des données de RS
	Cohérence-harmonisation	
	Volonté-leadership politique	
	Appropriation	
	Plaidoyer	
**La création et le maintien des ressources**	Capacités en recherche pour la santé (institutionnelles, humaines, leadership en recherche, autre)	L´existence d´universités avec des programmes de RS, d´instituts nationaux de RS, le nombre de chercheurs et de personnel de soutien impliqués dans la RS, les partenariats avec des institutions étrangères et la création de centres d´excellence
	Collaboration-partenariat-réseautage	
	Plaidoyer	
**La production et l´utilisation des connaissances**	Utilisation de la recherche	L´existence de plateformes d´échange de connaissances entre chercheurs et/ou décideurs politiques et PTF ainsi que l´existence de mécanismes de valorisation des résultats de la recherche
	Implication du secteur privé	
**Le financement de la recherche**	Implication du secteur privé Financement	L´existence d´une ligne dédiée à la recherche dans le budget du MSP comme indicateur du financement de la RS
	Financement	
	Plaidoyer	
**Autres codes utilisés**	Participation, engagement communautaire, crises de santé publique, contexte (économique, épidémiologique, géographique, politique, socioculturel, technologique)	

## Résultats

**Le contexte historique**: la recherche scientifique à Madagascar date de bien avant la période coloniale. Plusieurs documents basés sur des traditions orales et des manuscrits anciens font référence à l'existence de connaissances scientifiques et techniques dans la société traditionnelle malagasy entre les XVIe et XIXe siècles [16-19]. Toutefois, la colonisation par la France a marqué le début de l'institutionnalisation de la recherche à Madagascar avec la création d´instituts, tels que l´Institut Pasteur de Madagascar et l'Office de la Recherche Scientifique et Technologique d'Outre-Mer. Après l'indépendance du pays en 1960, la recherche scientifique est restée sous contrôle français avec des transferts progressifs de compétences aux chercheurs nationaux [20].

La structuration nationale de la recherche est véritablement amorcée avec la création en 1963 du “Secrétariat Général du Comité de la Recherche Scientifique et Technique”, chargé de planifier et de coordonner les activités de recherche dans le pays. Ces efforts de structuration sont interrompus par le mouvement nationaliste de 1972 qui a abouti à la révision des accords de coopération entre la France et Madagascar. Les assistants techniques présents sur l´île ont été rapatriés et remplacés par des cadres Malagasy peu préparés pour assurer la relève, et les financements pour la recherche provenant de la Métropole ont été suspendus [16,17]. La malgachisation du pays, c´est-à-dire l´usage de la langue Malagasy en lieu et place du français, a été instituée dans l'enseignement à tous les niveaux. D´après F. Blum, l'application inefficace de cette résolution a entraîné une mauvaise maîtrise des langues dans lesquelles la science est diffusée, notamment l'anglais et le français, renforçant ainsi l'isolement culturel et scientifique de Madagascar [20].

Ce mouvement a été suivi d´une longue période de crises socio-politiques qui ont contribué à freiner les tentatives de restructuration nationale telle que celle envisagée par le Plan d´Action Madagascar 2007-2012 [21], et entamé la confiance des partenaires au développement et des différentes institutions internationales vis-à-vis des institutions malgaches. Selon le plan de développement du secteur de la santé 2015-2019, le système de santé actuel se caractérise par une faible capacité à fournir des soins de santé à tous les niveaux, en raison du sous-financement de la santé, des ressources humaines insuffisantes, de l'obsolescence des infrastructures, et de l´accès limité aux soins [22]. L´épidémiologie locale est marquée par la persistance des maladies transmissibles et l´émergence des maladies non transmissibles qui affligent le système de santé d´un double fardeau. C´est dans ce contexte que le Gouvernement Malagasy s´efforce de mettre en place un SNRS qui réponde aux besoins de santé et développement des populations. Un sommaire des composantes du SNRS Malagasy est présenté dans le Tableau 3.

**Tableau 3:** sommaire des composantes du SNRS à Madagascar

Composantes du SNRS par les 4 fonctions		
**1) Gouvernance de la RS**	**Présent**	**Si oui, nom et (date de création)**
Existence Conseil National de Recherche pour la Santé	Non	Le Conseil National pour la Recherche pour la santé est en cours de création par le Ministère de la Santé Publique (MSP)
Existence d´un comité d´éthique	Oui	Le Comit d’Ethique de la Recherche Biomédicale de Madagascar (2016)
Structures de régulation/gouvernance	Oui	MESRS Direction Générale de la Recherche Scientifique ; MSP Direction des Etudes, Planification et Systèmes d´information (2019) ; Institut National de Recherche en Santé Publique (2019)
Lois et décrets d´orientation de la RS	Non	
Documents de politique, documents de stratégie	Oui	Stratégie nationale de la recherche scientifique à Madagascar (2013) ; Politique Nationale de Recherche pour la Santé (2016) Plan stratégique pour le développement de la recherche pour la santé à Madagascar 2018-2022 (2017)
Orientations stratégiques en recherche pour la santé, proposées dans la politique nationale du 2016	Oui	Renforcement des structures et mécanismes de leadership ; renforcement du partenariat ; Renforcement des structures, des mécanismes de production et de GIS et des bases factuelles sur la santé ; renforcement et promotion des capacités pour l´utilisation des TIC ; renforcement des mécanismes pour améliorer les capacités en RH ; renforcement des mécanismes pour un financement durable.
**2) Capacités en recherche pour la santé**		
Nombre de chercheurs temps-plein/million d´habitants % du PIB affecté à la recherche & le développement		24 hercheurs (FTE) par 1 million d´habitants ; 0.01%
Institutions publiques de recherche pour la santé	Oui	Centre National d´Application des Recherches Pharmaceutiques (1976), Institut National de Santé Publique et Communautaire (2002), Centre d´Infectiologie Charles Mérieux (CICM, 2011)
Institutions privées de recherche pour la santé/ONG	Oui	Institut Malgache de Recherche Appliquée (1957), Homeopharma (1992), Institut Pasteur de Madagascar (1898), ONG PIVOT (2013)
Autres institutions publiques de recherche qui interviennent dans la recherche pour la santé	Oui	Centre National de Recherche sur l´Environnement, Institut National des Sciences et Technologies Nucléaires (1976)
Universités avec faculté de médecine	Oui	Antananarivo (1960), Antsiranana, Fianarantsoa, Mahajanga, Toamasina et Toliara (1975)
Centres d´excellence	Non	
Laboratoires, équipement, et technologies disponibles	Oui	Laboratoires de l´Institut Pasteur de Madagascar, Laboratoire Rodolphe Mérieux d´Antanarivo
**3) Financement de la recherche pour la santé**		
Financement public	Non	
Financement international		
**4) Production et utilisation de la recherche pour la santé**	Oui	OMS, FNUAP, UNICEF, Banque Mondiale, Ambassade de France, Fondation Mérieux, etc.
Nombre de publications (2007-2018)		80 publications par million d´habitants soit 27 publications de 1er auteur par million d´habitants
Système d´information sanitaire performant	Non	
Plateformes de partage de l´information scientifique	Oui	1^ères^ Journées Scientifiques Nationales (Avril 2019) ; Conférences scientifiques ad-hoc (e.g. organisées par l´IPM, CICM)

**Les principaux facteurs qui influencent le système national de recherche pour la santé**: sur la base de ce contexte et de leurs expériences personnelles, les parties prenantes de cette étude ont identifié des enjeux et des facteurs cruciaux du développement de la RS à Madagascar. Nous présentons ces résultats à travers les différentes fonctions du SNRS.

**La gouvernance de la recherche pour la santé**: la RS à Madagascar est sous la double tutelle du Ministère de l´Enseignement Supérieur et de la Recherche Scientifique et du Ministère de la Santé Publique. Les mécanismes de coordination de la RS entre ces deux tutelles ne sont pas clairement définis. Selon les décideurs interrogés, le gouvernement travaille depuis plusieurs années à la mise en place d´une institution unique, le Conseil National de Recherche pour la Santé (CNRS) qui aurait pour rôle la régulation, la définition des responsabilités et la supervision de la RS dans le pays.

*« En fait, le premier obstacle, c´est l´interface unique qui manque. Il n´y a pas encore une structure de coordination pour la RS. On va mettre en place le CNRS. On attend la validation du texte.»* Décideur politique.

De l´avis de tous les informateurs, l´absence de coordination de la RS au niveau central semble être une faiblesse importante du SNRS avec de graves conséquences sur son fonctionnement, parmi lesquelles le clivage observé entre la recherche effectuée dans le pays et les priorités nationales de recherche. Toutefois, des expériences de coordination réussie ont été mentionnées, notamment dans le cadre de la coordination des activités de lutte contre le paludisme. En effet, le programme national de lutte contre le paludisme a mis en place à travers l´initiative Rollback Malaria, un dispositif de gouvernance comprenant des partenaires, des chercheurs et le personnel du programme. Ce mécanisme permet le partage d´informations et la prise de décisions, rapide, à tous les niveaux du système de santé.

Sur le plan réglementaire, il n´existe pas de loi d´orientation et de programmation de la RS dans le pays. Les chercheurs ont évoqué la nécessité de créer un cadre législatif définissant les rôles et responsabilités des différents acteurs du SNRS comme une étape importante pour le renforcement du SNRS. Madagascar fait partie des pays Africains ayant ratifié la déclaration d´Alger sur la RS en 2008 [9]. Cette déclaration fait force de loi dans le pays mais elle n´est renforcée par aucun texte juridique contextualisé qui en démontrerait l´appropriation. Le plan de développement du secteur de la santé 2015-2019 est focalisé sur le renforcement du système de santé dans ses missions de promotion et de prestation des services et ne propose pas explicitement des activités de RS [22]. Toutefois, la volonté politique du gouvernement à organiser la RS s´est illustrée en 2016 par l´élaboration et la validation de la politique nationale de la RS qui propose des orientations prioritaires dans ce domaine. Bien que cette politique ait été déclinée en un plan d´action budgétisé, une majorité d´informateurs jugent sa diffusion et sa mise en œuvre insuffisantes. En effet, seule une minorité de chercheurs et de PTF interrogés, était informée de l´existence de ces documents et plusieurs affirmaient n´avoir jamais été sollicités par le gouvernement pour appuyer leur mise en œuvre.

Globalement, les procédures d´implémentation des initiatives gouvernementales sont fastidieuses et souvent ralenties voire interrompues par le turn-over élevé des cadres de l´administration.

*« La continuité de l´État... c´est un mal chronique à Madagascar, nous parlons de discontinuité de l´État. On a planifié quelque chose mais lorsqu´un nouveau président arrive, on refait le monde. C´est un vrai problème! »* Chercheur.

Cependant, les chercheurs et décideurs interrogés reconnaissent la mise en place du Comité d´Ethique de la Recherche Biomédicale du Ministère de la Santé Publique (MSP) en 2016, qui fonctionne efficacement, comme un pas important dans la gouvernance éthique de la RS dans le pays.

**La création et le maintien des ressources**: la formation et la rétention de chercheurs compétents ont également été identifiées dans les entretiens comme des enjeux majeurs du SNRS Malgache. Malgré l´adoption du système Licence-Master-Doctorat, l´Université n´a pas encore migré vers une véritable formation à la recherche scientifique. Les chercheurs participants à l´étude ont souligné que ce déficit ne permet ni le développement d´une culture de recherche scientifique, ni l´émergence d'une génération de chercheurs hautement qualifiés. En outre, ils ont évoqué le fait qu´un nombre limité d´étudiants embrasse une carrière de chercheurs, pour des raisons liées aux conditions de travail et à l´absence d´un plan de carrière structuré pour les chercheurs. Les enseignants-chercheurs auraient tendance à s´investir plus dans des activités d´enseignement - plus lucratives et pérennes- que la recherche. De ce fait, les chercheurs compétents préfèrent parfois s´expatrier.

*«La capacité du pays à retenir ses chercheurs! Ceux qui ont les moyens, qui ont le niveau et qui veulent vraiment travailler dans la recherche vont ailleurs.»* Chercheur.

Un élément contextuel de démotivation des chercheurs qui a également été mentionné lors des entretiens est le manque de confiance des autorités politiques vis-à-vis des experts nationaux. Cette situation a été présentée comme une source de frustration qui ne concoure pas à la promotion de l´expertise locale.

*« Les dirigeants ont plus tendance à faire appel aux experts étrangers. C´est ça le gros problème. Il y a beaucoup d´experts à Madagascar mais souvent ils ne sont pas impliqués dans la prise de décisions.»* Chercheur.

Malgré toutes ces difficultés, les informateurs ont souligné le rôle important des partenariats internationaux dans le renforcement des capacités. En effet, les collaborations internationales sont parfois l´unique moyen pour certaines institutions de recherche d´accéder à des financements, d´améliorer leur plateau technique, ou d´intégrer des programmes de renforcement de capacités. Les collaborations avec les institutions bilatérales ou multilatérales semblent plus pérennes que les programmes verticaux, de courte durée, entre chercheurs. C´est le cas du partenariat entre Madagascar et l´Agence Internationale d´Energie Atomique (AIEA) décrit par un chercheur:

*« Entre l´AIEA et Madagascar, il y a un cadre de diffusion des connaissances et de transfert de technologies nucléaires. C´est une coopération technique qui est là depuis des décennies et continue à se développer. Chaque année, il y a 10 à 20 projets de transfert de technologies, transfert des connaissances à Madagascar. »* Chercheur.

**Le financement de la recherche**: à Madagascar le financement public de la RS est insignifiant comparé aux cibles normatives. D´après les informateurs, il n´existe pas une volonté politique clairement affichée de soutenir la RS, les discours politiques citent la recherche comme étant le levier du développement mais concrètement, peu de financements sont investis pour la soutenir.

*« Ils n´hésitent pas à dire que la recherche est la base du développement d´un pays. On proclame toujours qu´on veut faire de la recherche un appui au développement, mais est-ce que ça se traduit en actes et en financements ?»* Chercheur.

Les activités de recherche dans le pays sont essentiellement tributaires de la coopération internationale. Cette dépendance de la RS vis-à-vis des financements étrangers, créé un déséquilibre des forces entre les chercheurs locaux et leurs partenaires et rend difficile la pérennisation de ces activités. Comme l´a déclaré un des PTF:

*« Si le gouvernement américain arrête de financer la lutte contre le paludisme, cela représenterait environ 50% du budget. Le Fonds Mondial et d'autres petits donateurs représentent les 50% restants. Ce qui est investi par le gouvernement est extrêmement faible. Quand je pense à la pérennisation, je ne vois pas comment ce que nous faisons est soutenu en ce moment.»* PTF.

Outre la volonté politique, l´absence de financement public de la RS était perçue par certains informateurs comme la conséquence d´un déficit de leadership des chercheurs locaux qui auraient du mal à mener un plaidoyer efficace auprès des décideurs politiques pour une meilleure considération de la recherche dans les priorités gouvernementales. Dans le même ordre d´idées, certains PTF interrogés ont déploré la faible sollicitation de leurs services par les chercheurs et le MSP, et la capacité limitée de ceux-ci à mobiliser les opportunités de financements existantes, auprès des PTF ou du secteur privé.

*« Tout dépend de la volonté, du leadership national. Si le ministère ou l´Institut National de Santé Publique et Communautaire nous sollicitent pour appuyer un thème de recherche quelconque, on est là pour cela. Mais il n´y pas d´initiative. »* PTF.

Certaines structures de recherche parviennent toutefois à obtenir des financements localement, notamment du secteur privé. L´Institut National des Sciences et Techniques Nucléaires (INSTN) par exemple, a réussi à développer des partenariats avec des industriels pour la gestion des déchets radioactifs des mines et un appui technique dans la recherche. Ce partenariat permet à l´INSTN de mettre leur expertise au service de l´industrie tout en générant des fonds de recherche et en protégeant la santé des populations.

**La production et l´utilisation de la recherche pour la santé**: quoiqu´en progression, la production scientifique à Madagascar reste faible. Partant d´environ 20 publications référencées en 1987, le nombre de publications est passé à 80 en 2006 et 188 en 2014 [12]. Cette performance classe Madagascar parmi les pays qui ont la plus faible productivité scientifique dans le monde. Il existe cependant à Madagascar, des revues scientifiques nationales qui paraissent sporadiquement mais ne sont pas référencées dans les bases de données bibliographiques internationales. Plusieurs fora *ad-hoc* de dissémination des résultats de recherches scientifiques existent dans le pays, organisés par le MSANP (1^e^ journées scientifiques de Madagascar en 2019), des institutions de recherche comme l´Institut Pasteur de Madagascar, ou des PTF. De l´avis des chercheurs interrogés, les résolutions issues de ces rencontres et importantes pour le renforcement du SNRS ne sont malheureusement pas souvent implémentées par les décideurs. Le pays ne dispose pas d´un système d´information sanitaire performant, ce qui freine la capacité du gouvernement à générer les données indispensables pour le suivi de l´évolution de leurs indicateurs prioritaires de santé. De plus, les secteurs économiques et industriels ne sont pas suffisamment sollicités pour aider à la valorisation de la RS. Quoique marginaux, des exemples de valorisation existent, notamment dans les domaines des technologies nucléaires et de la phytothérapie.

## Discussion

Les facteurs qui influencent le développement des fonctions du SNRS malagasy sont multiformes. D´une part, la gouvernance de la RS s´organise peu à peu avec plusieurs institutions de recherche qui opèrent malheureusement sans un cadre législatif spécifique et sans mécanisme de coordination. D´autre part, les ressources humaines pour la RS sont globalement insuffisantes, dû à des défis dans la formation et la rétention des chercheurs. Sans financement public adéquat, l´intégralité des fonds pour la RS provient des bailleurs internationaux, ce qui veut dire avec leurs programmes et leurs priorités. Le soutien pour la rédaction scientifique ancrée dans la formation universitaire est faible, de ce fait, la production et l´utilisation de la recherche se limitent à des occasions ad-hoc et ne sont pas systématiquement mises en lien avec les besoins sanitaires et les programmes nationaux. Cette analyse du SNRS a permis d’identifier deux thèmes majeurs comme étant des facteurs particulièrement importants dans l´évolution de la RS dans le pays: la collaboration internationale et le leadership et/ou l´engagement des décideurs.

**Le rôle des collaborations internationales**: les collaborations scientifiques internationales influencent plusieurs fonctions des SNRS en Afrique. En effet, les premiers instituts de recherche ont été implantés dans la plupart des pays en développement grâce à des accords de coopération plus ou moins équitables avec des pays occidentaux. Ces coopérations ont un pouvoir structurant sur les plan individuel, institutionnel et national en ceci qu´elles offrent aux pays du Sud, des moyens pour une mise à jour des connaissances, des méthodes et techniques scientifiques [23]. C´est le cas à Madagascar où les coopérations scientifiques constituent la principale source de financement de la recherche et de renforcement des capacités. Les coopérations qui impliquent les gouvernements locaux, qui sont établies dans une perspective cohérente avec les besoins du pays et sur le long terme semblent être les plus adaptées pour le renforcement du SNRS [24]. Toutefois, mal négociées, ces collaborations peuvent créer un déséquilibre de forces qui aboutit à un clivage entre la RS menée dans le pays et les besoins prioritaires du pays, l´agenda de la recherche étant dicté par ces partenaires internationaux, conformément à leurs priorités et leurs programmes. Ainsi, les chercheurs et décideurs Malagasy doivent pouvoir mener une discussion décomplexée avec les PTF pour établir les principes directeurs de partenariats de recherche équitables dans le pays. C´est déjà le cas dans certaines institutions de recherche. Par exemple, les collaborations internationales portant sur l´étude des plantes médicinales avec le CNARP, sont conditionnées par l´implication d´un chercheur national à toutes les étapes du processus d´identification du principe actif, jusqu´au dépôt des brevets, le cas échéant. Plusieurs autres modèles de collaboration Nord-Sud équitables et fructueux dont Madagascar pourrait s´inspirer existent [14,25,26]. Par exemple, Kok *et al*. ont rapporté l´expérience développée par les gouvernements Ghanéen et Néerlandais entre 2001 et 2008 [25]. Cette approche de collaboration avait pour mission de soutenir une recherche axée sur la demande locale et dirigée par des chercheurs nationaux. L´évaluation de ce programme a montré que 79 études proposées par des chercheurs nationaux avaient été financées et que les politiques nationales de santé avaient été positivement impactées par les résultats de ces études.

**Le rôle des décideurs politiques et institutionnels**: la mise en place d´un SNRS solide, capable de soutenir le système de santé en proposant des solutions efficaces et pérennes aux défis sanitaires du pays relève de la responsabilité de l´Etat. L´absence de textes législatifs et de financement public, la non-maîtrise de l´agenda de la RS, les efforts inaboutis de coordination et le peu d´investissement observé dans la formation et la valorisation des chercheurs sont des indicateurs de la faible volonté de l´Etat à promouvoir la RS à Madagascar. Cette situation est commune à plusieurs pays en développement qui, face à des défis multiformes ont érigé la RS au rang des priorités négligées [24,27]. Plusieurs études ont identifié les problèmes de gouvernance, l´instabilité politique, l´ignorance par les décideurs de l´intérêt de la recherche pour le développement et l´absence de culture d´évaluation, comme étant des barrières structurelles au développement de la RS en Afrique [5,6,28]. Une analyse rigoureuse des capacités de gouvernance et de régulation de la recherche permettrait effectivement d´identifier et d´infléchir les obstacles spécifiques au contexte Malagasy. Des plateformes d´échanges entre chercheurs et décideurs doivent être développées pour renforcer la compréhension par ces derniers de l´importance de la RS et promouvoir l´utilisation des données contextuelles pour la prise des décisions. Compte-tenu du niveau de performance actuel du pays en RS, la volonté politique pourrait également s´illustrer par un investissement sérieux dans le développement des infrastructures et la formation des futurs chercheurs. Pour ce faire, l´Etat pourrait entre autres, solliciter l´implication de la diaspora scientifique Malagasy et son réseau de collaborations. La maîtrise de l´agenda de la RS du pays passe inévitablement par des efforts de coordination mais aussi par la mise à disposition d´un financement public de la recherche. A cet effet, les décideurs doivent faire preuve d´innovation. Plusieurs pistes ayant fait leurs preuves dans des contextes similaires peuvent être exploitées, telle que la taxation de la téléphonie mobile, des boissons alcooliques ou des matières premières [18,19]. L´implication du secteur privé dans le financement de la RS contre des réductions de taxes ou d´autres compensations pourrait également être envisagée.

## Conclusion

Cette étude a présenté un inventaire des composantes du SNRS malagasy tout en analysant les facteurs qui influencent son évolution. La volonté et la crédibilité politiques doivent s´illustrer par la mise en place de fonds publics pour la recherche et la mise en fonction des structures de coordination en instance de création, pour fédérer les capacités de recherche existantes dans le pays. Les partenariats avec des structures internationales sont les principaux pourvoyeurs de ressources. Les chercheurs et décideurs malagasy devront continuer de déployer leur leadership et faire preuve d´audace et de créativité pour renforcer les partenariats existants, en développer de nouveaux et négocier des conditions de mise en œuvre équitables.

## Remerciements

Les auteurs tiennent à remercier tous les participants à cette étude, particulièrement M. Aina Rakotobe qui a largement contribué à l´organisation du travail sur le terrain, ainsi que les autres membres de l´équipe du projet qui ont collaboré à la conception des outils de collecte et d´analyse des données et dont les commentaires ont contribué à enrichir ce travail. Financement: cette recherche a été effectuée dans le cadre du projet *« Building the case for investment in health science research in Africa »*financé par le Wellcome.
